# Uncovering Pet Issues: A Survey of Professionals Working With Older Adults and Care Partners

**DOI:** 10.1093/geroni/igab046.832

**Published:** 2021-12-17

**Authors:** Jessica Bibbo, Justin Johnson, Jennifer Drost, Margaret Sanders

**Affiliations:** 1 Benjamin Rose Institute on Aging, Cleveland, Ohio, United States; 2 Summa Health System, Akron, Ohio, United States; 3 Northeast Ohio Medical University, ROOTSTOWN, Ohio, United States

## Abstract

Pets can play an important role in older adults’ health behaviors and decisions. However, the degree to which these issues are encountered or addressed by professionals working with this population remains unknown. An interdisciplinary (e.g., healthcare, social services) sample of professionals (N=72, 93.05% female, Mage=48.82, SDage=12.57) completed an online survey focused on the pet ownership issues they have encountered while working with older adults, persons with dementia, and care partners. The professionals (n=66) estimated 42.86% of their clients had been pet owners, and 45.58% regularly asked their clients about pets. Issues raised to the professionals varied by type of client. Older adults most often brought up exercising the pet, routine veterinary care, and the financial aspect of ownership (all 37.50%). Persons with dementia most often discussed accessing pet care items (12.50%), exercising the pet (9.72%), and basic pet care (8.33%). Care partners brought up basic pet care (33.33%), planning for the pet due to their care recipients’ housing transition (26.38%), and exercising the pet (25.00%). Professionals reported talking to clients about planning for the pet due to housing transition, concerns about falling, and concerns about the pet’s behavior (all 31.94%). The professionals (n=69) were very favorable toward pet ownership in general (M=4.43, SD=0.78) (1=extremely unfavorable, 5=extremely favorable), less favorable about older adult pet ownership (M=4.15, SD=0.72, p=.002), and even less favorable about persons with dementia owning pets (M=3.51, SD=0.93, p<.001). The results provide evidence that pet ownership issues are likely encountered in geriatric service settings and may shape healthy aging.

